# Characteristics of macular morphology and microcirculation in diabetic macular edema patients with serous retinal detachment

**DOI:** 10.1186/s12886-022-02523-7

**Published:** 2022-07-11

**Authors:** Min Xu, Huirong Xu, Xiao Li, Fang Chen

**Affiliations:** Department of Ophthalmology, Subei People’s Hospital affiliated to Yangzhou University, Yangzhou, China

**Keywords:** Diabetic macular edema, Serous retinal detachment, Macular morphology, Macular microcirculation

## Abstract

**Background:**

To analyze and compare the characteristics of macular morphology and microcirculation in diabetic macular edema (DME) patients with and without macular serous retinal detachment (SRD).

**Methods:**

One hundred eyes in 81 patients diagnosed with the DME (the central macular thickness (CMT) of ≥ 300 μm) from March 2020 to November 2020 were selected. According to whether complicated with SRD, patients were divided into DME with SRD (60 eyes) and without SRD (40 eyes) groups. We analyzed the following parameters: CMT, central retinal thickness (CRT), subfoveal choroidal thickness (SFCT), number of hyperreflective foci (HF) in the complete retina, inner retina, outer retina, and subretinal space, the integrity of the ellipsoid zone (EZ) and external limiting membrane (ELM), the presence of disorganization of inner retinal layers (DRIL), foveal avascular zone (FAZ) area, and the vascular flow density of superficial capillary plexus (SCP), deep capillary plexus (DCP), and choriocapillaris.

**Results:**

(1) Compared to the group without SRD, the group with SRD had a greater CMT (*P* < 0.05) and a smaller CRT (*P* < 0.001); (2) The number of the HF in the complete retina, outer retina, and the subretinal space was larger in the group with SRD (*P* < 0.001); 3.The proportion of the EZ disruption (*P* < 0.05) and ELM disruption (*P* < 0.001) were higher in the group with SRD; 4. The SFCT (*P* < 0.05) and the vascular flow density of choriocapillaris (*P* < 0.05) were greater in the group with SRD; 5. There were no significant differences in the FAZ area and the vascular flow density of the DCP and SCP (*P* > 0.05); 6. The presence of the SRD was correlated with the integrity of the ELM, the number of HF in the complete retina, outer retina, and subretinal space (*χ2* = 26.930, *OR* = 0.707, 0.263, 0.995, *P* < 0.001), as well as the SFCT (*OR* = 0.992, *P* < 0.05).

**Conclusions:**

The results support the hypothesis that the presence of the ELM disruption, the larger number of the HF, and the thickening and hyperperfusion of the choroid may be involved in the pathogenesis of SRD in DME.

## Background

Diabetic macular edema (DME) is a complication of diabetes and one of the major causes of vision loss in patients with diabetes mellitus (DM) [1]. This condition can occur in all stages of diabetic retinopathy (DR) and considerably influences the quality of a patient’s life. In recent years, with the emergence of anti-VEGF drugs, the prognosis of the DME has been significantly improved in many patients, but a barely satisfactory therapeutic effect has been observed in some patients [2].

Based on the morphology acquired from the optical coherence tomography (OCT), the DME exhibits the following structural changes: sponge-like diffuse retinal thickening (SDRT), cystoid macular edema (CME), and serous retinal detachment (SRD). On the OCT images, the SRD was manifested as an opaque dark area of fluid between the neurosensory retina and the retinal pigment epithelium (RPE), which accounts for approximately 15–30% of DME patients [3]. Previous research found poorer therapeutic effects and visual outcomes from anti-VEGF drug treatment than the corticosteroids therapy in DME patients with SRD [4]. Therefore, this study aimed to explore the pathogenesis of SRD in DME. Previous evidence revealed that the leakage of fluid from the destruction of the blood-retinal barrier and the high perfusion of the choroidal vessels outweighed the reabsorption capacity of the RPE cells. This perturbation might be the primary factor for the pathogenesis of SRD [5]. Furthermore, an increased level of inflammatory cytokines such as interleukin-6 (IL-6) was found in the aqueous humor and vitreous fluid of patients with SRD, suggesting that this condition might be associated with the inflammation [6]. Currently, the pathophysiology of the SRD in DME and its relationship with microvascular damage of the retina and choroid remain speculative.

Optical coherence tomography angiography (OCTA) is a safe and quick noninvasive approach to obtain images of vessels of the retina and choroid in situ. The OCTA quantitatively evaluates the different layers of the retinal capillary network, which offsets the disadvantages of using fundus fluorescein angiography (FFA) in the evaluation of the flow in the DR and DME. Hence, a standard of reference can be established for disease evaluation and prognostic prediction. Therefore, the present study aimed to analyze and compare the characteristics of the macular morphology and microcirculation by OCTA in two types of DME eyes: a group with SRD and a group without SRD. Further, this study planned to evaluate the characteristics of the DME eyes with SRD carefully to dissect out the pathogenesis of SRD.

## Methods

### Participants

Informed consent was obtained from each patient, and the research was carried out in compliance with the Declaration of Helsinki principles. The patients with the DME were retrospectively assessed from March 2020 to November 2020 from the records in the Ophthalmology Department of Subei People’s Hospital (Yangzhou, Jiangsu, China). The stages of pre-proliferative diabetic retinopathy (PPDR) or proliferative diabetic retinopathy (PDR) were determined by FFA, and the DME was established by the OCT/OCTA examination. The DME was defined as a central retinal thickness of ≥ 300 m with the presence of intraretinal or subretinal fluid within the range of 1 mm of the macular fovea [7]. The exclusion criteria were as follows: [1] Patients complicated with uncontrolled hypertension (systolic blood pressure of > 140 mmHg and diastolic blood pressure of > 90 mmHg); [2] Subjects with other retinal diseases (e.g., retinal vein occlusion, macular epiretinal membrane, and vitreomacular traction syndrome); [3] The conditions accompanied by other systemic diseases that may affect the fundus (e.g., hematologic diseases, autoimmune diseases, neurodegenerative diseases, severe kidney diseases or chronic renal failure); [4] Patients with a history of anti-VEGF or dexamethasone intravitreal injection, grid macular laser or retinal photocoagulation within six months, and a history of a vitrectomy or cataract surgery within three months; [5] Poor OCTA images with a signal strength index of < 6 due to the media opacity or significant motion artifact. To evaluate the characteristics of the DME eyes with SRD carefully to dissect out the pathogenesis of SRD, patients were divided into two groups depending on their conditions of whether complicated with SRD artificially. Finally, a total number of 81 cases (100 eyes) were included in the study: 47 cases (60 eyes) with SRD and 34 cases (40 eyes) without SRD. The combined SRD group consisted of the simple SRD, CME combined with SRD, and SDRT combined with SRD; the non-combined SRD group included the type of simple CME and simple SDRT.

## Methods

### General data

The systemic characteristics of patients, including sex, age, diabetic duration, the value of blood glucose (GLU), glycated hemoglobin A1c (HbA1c), and creatinine were evaluated.

#### Clinical examinations

**Fig. 1:**
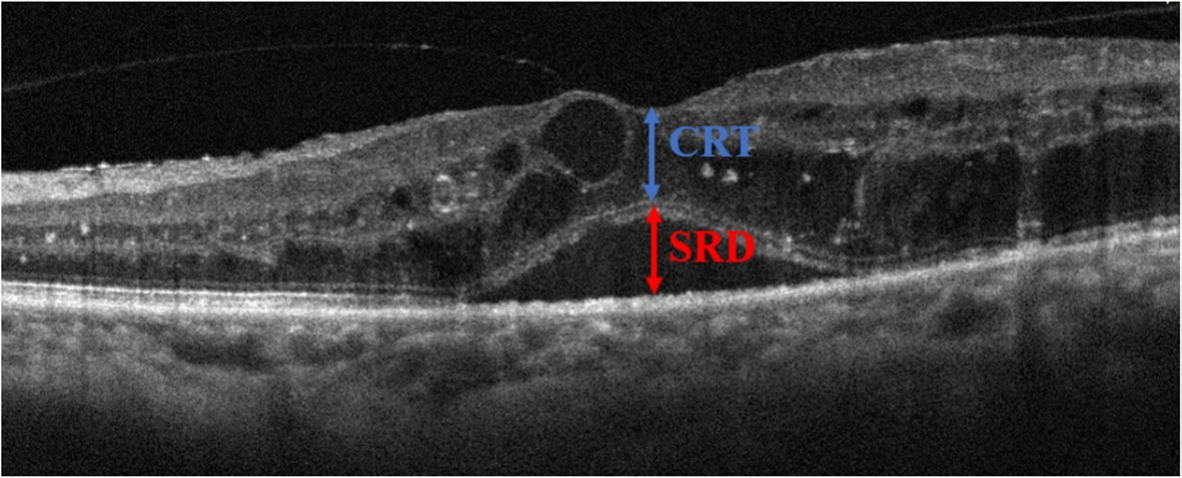
The OCT scan image of a patient with SRD. The red arrow indicates the height of the SRD, and the blue arrow indicates the height of the CRT (the height of the CMT-SRD)

All patients were subjected to scanning by OCT and OCTA (RTVue XR; Optovue, Fremont, CA, USA). The CMT, defined as the average distance between the inner limiting membrane of the retina and the RPE within 1.0 mm of the macular fovea, was computed using OCT mapping software. The heights of the SRD and central retinal thickness (CRT) were measured in the group with SRD (Fig. 1). SRD height, defined as the the length between the outer retinal surface and RPE, was measured manually with the caliper function of the OCT device. The CRT was obtained by the CMT minus the SRD height. The SFCT, defined as the vertical distance from the Bruch membrane under the fovea to the highly reflective light band on the inner surface of the sclera, was an average value of the results measured manually with the caliper function of the OCT device by two ophthalmologists. The integrity of the ellipsoidal zone (EZ) and external limiting membrane (ELM), the presence of the disorganization of the inner retinal layers (DRIL) was also judged by two ophthalmologists, if the results are inconsistent, it will be read and recorded by the chief retinal specialist. The number of hyperreflective foci (HF) was also an average value of the results counted by two ophthalmologists. The integrity of the EZ and ELM was denoted as the integrity within 3.0 mm of the central fovea. The DRIL was described as the disorder of the boundary among the ganglion cells, the inner plexiform layer, the inner nuclear layer, and the outer plexiform layer, as previously reported [8]. The HF was defined as discrete and well-circumscribed dots with the same or higher reflectivity as RPE within the central 3.0 mm zone around the fovea [9]. The number of HF in the inner retina (from the internal limiting membrane to the outer nuclear layer), the outer retina (from the external limiting membrane to the outer segment of photoreceptor), and the subretinal space (from the outer segment of the photoreceptor to the RPE) were recorded, respectively. The foveal avascular zone (FAZ) area, vascular flow density of superficial capillary plexus (SCP)/deep capillary plexus (DCP), and choriocapillaris were was computed using OCTA mapping software. The SCP was specified as the capillary plexus from 3 to 15 μm below the inner plexiform layer, and the DCP was specified as the capillary plexus from 15 to 70 μm below the inner plexiform layer [10]. The boundary between the SCP and DCP was adjusted manually in case of incorrect segmentation. The vascular flow density of the SCP/DCP was interpreted as the ratio of the flow area within 3 × 3 mm^2^ of the fovea to the scanning area. The FAZ area was denoted as the non-vascular region surrounded by the continuous capillary plexus of the retina. The vascular flow density of choriocapillaris was categorized as the ratio of the flow area within a diameter of 2 mm centered on the fovea to the scanning area.

### Statistical analysis

All statistical analyses were performed using the SPSS software, version 25.0 (IBM Co., Armonk, NY, USA). The measurement data were tested for normality. Continuous variables are presented as mean *±* standard deviation (*𝑥̅±s*), whereas the measurement data that did not conform to the normal distribution are expressed by the median (quartile interval). The measurement data were analyzed by independent *t*-test or non-parametric rank-sum test, and the counting variables were verified by the chi-square test. According to the data type, the chi-square test, logistic regression analysis, and the Spearman correlation analysis were performed for the correlation analysis. *P-*value of < 0.05 was considered to define statistically significant differences.

## Results

### Comparison of general data

There were no significant differences in sex, age, best-corrected visual activity (BCVA), course of diabetes, HbA1c, and creatinine between the two groups (*P* > 0.05). In the 60 eyes with SRD, there were 40 eyes (66.7%) in the stage of PPDR and 20 eyes (33.3%) in the stage of PDR. Of the 40 eyes without SRD, 28 eyes (70.0%) were in the stage of PPDR and 12 eyes (30.0%) were in the stage of PDR. There was no significant difference in the proportion of PPDR and PDR between the two groups (Table 1).

**Table 1:** Comparison of the baseline characteristics between the two groups of patients

	DME with SRD	DME without SRD	*U/ t /χ2*	*P*
Sex			0.24	0.624^c^
Male	30 (50.0%)	22 (55.0%)		
Female	30 (50.0%)	18 (45.0%)		
Age	56.37 ± 9.97	54.84 ± 8.04	0.810	0.420^a^
LogMAR BCVA	0.52 (0.40, 0.70)	0.52 (0.30, 0.79)	885.500	0.776^b^
Course of the DM (year)	10.00 (6.50, 10.00)	10.00 (5.50, 18.50)	1084.500	0.378^b^
HbA_1c_(%)	7.80 (7.30, 8.20)	7.40 (6.90, 7.80)	937.500	0.058^b^
Creatine(µmol/L)	79.00 (68.50, 83.50)	77.00 (65.50, 92.50)	1088.000	0.398^b^
Stage			0.123	0.726^c^
PPDR	40 (66.7%)	28 (70.0%)		
PDR	20 (33.3%)	12 (30.0%)		

^a^independent *t*-test; ^b^Mann-Whitney *U* test; ^c^Chi-square test

### Comparisons of the CMT, CRT, and SFCT between the two groups

There were statistically significant differences in terms of CMT [487.00 (392.00, 595.00) µm] vs. [403 (356.00, 463.50) µm] (U = 765.500, *P* = 0.002) and SFCT [ (296.75 ± 53.41) µm vs. (271.83 ± 56.66) µm] (t = 2.231, *P* = 0.028) between the group with SRD and the group without SRD. On the other hand, the CRT of 403.00 (356.00, 463.50) µm in the group without SRD was larger than that in the group with SRD [294.05 (228.25, 402.50) µm] (*P* < 0.001) .

### Comparison of the EZ and ELM, and the presence of DRIL between the two groups

The proportion of the EZ and ELM disruption in the group with SRD was significantly higher than that in the group without SRD. EZ disruption: (96.7%) vs. (82.5%), (χ2 = 4.279, *P* < 0.039); ELM disruption: (81.7%) vs. (30.0%), (χ2 = 26.930, *P* < 0.001). The DRIL occurrence in the group without SRD (87.5%) was significantly higher than that in the group with SRD (60.0%), (*χ2* = 8.815, *P* = 0.003) .

### Comparison of the HF between the two groups

The numbers of the HF in the full retina [10.00 (8.00, 13.00)], the outer retina [3.00 (2.00, 4.00)], and the subretinal space [1.00 (0.00, 2.00)] were larger in the group with SRD than in the group without SRD, which were 5.50 (3.25, 8.00), 0.00 (0.00, 1.00), 0.00 (0.00, 0.00), respectively (U = 368.000, 238.500, 486.000; *P* < 0.001). There was no significant difference in the number of the HF in the inner retina between the two groups (U = 824.500, *P* = 0.070).

### Comparison of the FAZ area, vascular flow density of the SCP, DCP, and choriocapillaris between the two groups

No significant difference was found in the FAZ area and the vascular flow density of the SCP and DCP between the two groups. However, the vascular flow density of the choriocapillaris in the group with SRD (53.63% ± 6.87%) was significantly greater (t = 2.112, *P* = 0.037) than that in the group without SRD (50.81% ± 5.28%) (Table 2).

**Table 2:** Comparison of the FAZ area, vascular flow density of the SCP, DCP, and choriocapillaris between the two groups

	DME with SRD	DME without SRD	*U/ t*	*P*
SCP (%)	36.29 ± 5.47	35.23 ± 5.19	0.938	0.350^a^
DCP (%)	40.69 ± 5.93	40.69 ± 5.99	-0.032	0.974^a^
FAZ area(mm^2^)	0.31 (0.22, 0.40)	0.38 (0.29, 0.58)	381.500	0.284^b^
Choriocapillaris (%)	53.63 ± 6.87	50.81 ± 5.28	2.112	0.037^a^

^a^independent t-test; ^b^Mann-Whitney *U* test

### Correlation analysis

The ELM was intact in 11 eyes (18.3%) but disrupted in 49 eyes (81.7%) in the group with SRD (60 eyes). On the other hand, of 40 eyes without SRD, the ELM was intact in 28 eyes (70.0%) but disrupted in 12 eyes (30.0%). The SRD was significantly correlated with the integrity of the ELM (χ2 = 26.930, *P* < 0.001). The presence of the SRD was significantly correlated with the number of the HF in the complete retina, the outer layer, and the subretinal retina (*OR* = 0.707, 0.263, 0.995, *P* < 0.001) but was not correlated with the number of the HF in the inner retina (*OR* = 0.884, *P* = 0.066). There was a significant correlation between the presence of the SRD and SFCT (OR = 0.992, *P =* 0.031). In the DME eyes with SRD, the height of the SRD was correlated with the number of the HF in the complete retina and outer retina (r = 0.404, 0.346, *P =* 0.001, 0.007). No significant correlation was found between the height of SRD and the number of the HF in the subretinal space, as well as the SFCT (*r* = 0.182, 0.232, *P =* 0.165, 0.075) (Table 3).

**Table 3:** Correlation analysis

	Integrity of ELM		HF in full retina		HF in outer retina		HF in subretinal layer		SFCT	
	*χ*^2^	*P*	*OR*	*P*	*OR*	*P*	*OR*	*P*	*OR/r*	*P*
srd presence	26.930	< 0.001^a^	0.707	< 0.001^b^	0.263	< 0.001^b^	0.995	< 0.001^b^	0.992	0.031^b^
srd height	-	-	0.404	0.001^c^	0.346	0.007^c^	0.182	0.165^c^	0.232	0.075^c^

^a^ Chi-square test, ^b^ Logistic regression analysis, ^c^ Spearman correlation analysis

## Discussion

The pathogenesis of DME is complex. Currently, the destruction of the blood-retinal barrier and the RPE barrier has been considered to be closely related to the occurrence of the DME [11]. A disruption in the retinal barrier caused exudation of the fluid and protein and their accumulation between the outer plexiform layer and the inner nuclear layer of the retina, resulting in the DME. In addition, a variety of cytokines such as VEGF and IL-6 have been involved. Reports from the histopathological study revealed that the SDRT type was closely related to the dysfunction of the Müller cells [12]. The CME type was considered to have resulted from the gradual destruction of the Müller cells that led to the liquefactive necrosis and cystic dark cavity formation. While the SRD type was different from the former two, the dysfunction of the RPE barrier and the disruption of ELM may have been responsible for that outcome [13]. The prognosis of visual acuity in the SRD type DME was better when the treatment with corticosteroids was administered rather than with anti-VEGF [4]. The pathophysiology of the SRD and its relationship with the microvascular damage of the retina and the choroid are still speculative. To explore the possible pathogenesis of the SRD in the DME patients with SRD, this study analyzed and compared the differences of the macular morphology and microcirculation in the DME patients with and without SRD. Different viewpoints exist concerning the macular flow in patients with the DR or DME. Previous studies showed that the vessel density of the retina was related to the progression of the disease [14]. The lower vessel density was often related to more serious DR, and the DCP was more likely to be affected by ischemia than the SCP. In this respect, Pongsachareonnont et al. [10] analyzed the differences in the FAZ area of the superficial and deep capillary plexus between 41 non-proliferative diabetic retinopathy (NPDR) eyes and 42 PDR eyes with DME. The study found that the FAZ in the deep capillary plexus was larger in the PDR eyes. Additionally, the study investigated the differences in the microcirculation between the eyes of the NPDR and PDR without the DME but found no significant differences, which indicated that the macular vascular density may have been related to the progression of DR and the complication by the DME. Lee et al. [15] found that the eyes with the DME had lower vascular density and larger FAZ area in the DCP compared to the non-DME eyes. Furthermore, it revealed that the larger damage of the integrity of the DCP and the FAZ area seems to have a poorer response to anti-VEGF treatment. comparing of affected eyes with a good or poor response to anti-VEGF treatment The DME was often classified according to the OCT patterns, however, a categorization based on the complication with the SRD has been limited. This study results showed no prominent difference in the FAZ area and the vessel density of the SCP and DCP between patients with SRD and patients without SRD. We considered that these results may have been related to the insignificant difference in the severity of the DR among eyes included in the study, resulting in similar damage to the SCP and DCP. These findings may indicate that the macular vessel density in patients with the DME may have been more closely associated with the severity of the DR. Nevertheless, larger sample size is required to precisely analyze its associations with SRD. The results of this study showed that the SFCT was thicker in the group with SRD than in that without SRD, with a significant correlation between the presence of SRD and SFCT. Increased SFCT might have elevated the likelihood of the SRD occurrence. However, no significant correlation was established between the height of the SRD and SFCT, which was consistent with the results of many of the previous elucidations [16]. It may be speculated that the occurrence of the SRD may be related to the thickening of the choroid. However, further studies are needed to thoroughly investigate this hypothesis. The study by Campos et al. [17] showed that the decrease in alkaline phosphatase activity and the release of nitric oxide in the choriocapillaris of patients with the DR induced the inflammation and ischemia of the choroid, which destroyed the blood-retinal barrier and led to the formation of the SRD. Although the density of the choriocapillaris could not fully reflect the state of the choroid, it provided a basis for a speculation to see whether the presence of SRD may be related to the hyperperfusion state of the choroid. Therefore, the potential correlation between the different DME types and the choroidal thickness needs to be further explored.

The ELM was considered a barrier restricting the movement of macromolecules formed by the bridging connection between the adjacent photoreceptors and the Müller cells. The ELM limited the migration of the exudated lipids, proteins, and other macromolecules from the inner layer of the retina to the outer layer [18]. Some scholars proposed that the ELM disruption may be a contributing factor to the formation of the SRD. A previous histopathological study found that the presence of the SRD was related to the disruption of the ELM, and the subretinal effusion may have been derived from the exudates that flew into the subretinal space through the discontinuous ELM [13]. The results of this investigation further supported this theory. This study found that the extent of the ELM disruption in the DME patients with SRD was higher than that in the patients without SRD (*P* < 0.001). Besides, there was a significant correlation between the presence of the SRD and the integrity of the ELM.

Recently, increasingly more attention has been paid to the role of the HF from the OCT images as an imaging biomarker in vitreoretinal diseases. The HF was believed to be the product of lipid exudation after the destruction of the blood-retinal barrier [19]. Other elucidations speculated that it was the degenerated photoreceptor cells or microglia activated by inflammation; some scholars even considered that the HF was the RPE cells migrating to the retinal neuroepithelial layer since the reflex intensity of HF was the same as that of the RPE cells [20]. Notably, Vujosevic et al. [4] found that the eyes with more HF at the baseline were more effective in the treatment of dexamethasone implants, indicating that the larger number of HF may have been related to the local active inflammatory response. Furthermore, there was a direct and significant correlation between the number of HF and the occurrence of the SRD in the DME eyes, which further validated the hypothesis that an inflammatory reaction might have been a key factor in the pathogenesis of the DME with SRD. The HF was also considered to be the precursor of hard exudation, and the more HF found in each layer of the retina resulted in a worse visual acuity following the anti-VEGF treatment; the location of the HF may have also been a major factor affecting the prognosis of the DME [21]. This study results showed that the number of HF in the outer retina, subretinal space, and complete retina in the group with SRD was larger than that in the group without SRD. Further, correlation analysis revealed that the higher number of the HF in the outer retina, subretinal space, and complete retina increased the likelihood of the SRD occurrence, and the larger number of HF in the outer and complete retina elevated the height of the SRD. Therefore, we speculated that the formation of the SRD may have been related to the presence of a certain number of HF.

The EZ corresponds to the ellipsoid of the inner segment of the photoreceptor cell, which is rich in mitochondria. Thus, the disruption of the EZ affects the energy supply of the photoreceptor cells and hence the visual function. The DRIL is a structural disorder of the inner retina. Previous studies have confirmed that the FFA showed macular ischemia in the corresponding region of the DRIL. In recent years, many investigations have shown that the disruption of the EZ and the presence of the DRIL could be used as the major indicators of the visual outcome in the DME [8, 22]. In the present study, it was found that the proportion of the EZ disruption in the group with SRD (96.7%) was significantly higher than that in the group without SRD (82.5%), which was in agreement with the findings of the previous elucidations. However, the proportion of the DRIL in the group without the SRD was significantly higher than that in the group with the SRD, and the CRT in the group without the SRD was significantly higher than that in the group with the SRD. These outcomes might have been due to the larger number of eyes with the CME in the group without SRD. However, the validation of the response from these two groups to the VEGF treatment needs further follow-up.

## Conclusions

In this study, by comparing the parameters of macular morphology and microcirculation of the DME patients with and without SRD, we found that the disruption of the ELM, the larger number of the HF, and the thickening and hyperperfusion of the choroid may have been related to the formation of the SRD in the DME. These current findings contribute to the existing understanding of the pathogenesis of the SRD in DME eyes.

## Data Availability

The datasets used and/or analyzed during the current study are available from the corresponding author on reasonable request.

## Abbreviations

CRT: Central retinal thickness
CMT: Central macular thickness
CRT: Central retinal thickness
CST: Central subfield thickness
DCP: Deep capillary plexus
DM: Diabetes mellitus
DME: Diabetic macular edema
DR: Diabetic retinopathy
DRIL: Disorganization of inner retinal layers
FAZ: Foveal avascular zone
FFA: Fundus fluorescein angiography
ELM: External limiting membrane
EZ: Ellipsoid zon
HF: Hyperreflective foci
OCT: Optical coherence tomography
OCTA: Optical coherence tomography angiography
PDR: Proliferative diabetic retinopathy
PPDR: Pre-proliferative diabetic retinopathy
SCP: Superficial capillary plexus
SDRT: Sponge-like diffuse retinal thickening
SFCT: Subfoveal choroidal thickness
SRD: Serous retinal detachment
